# Crystal structure of 3-hy­droxy-2-(4-hy­droxy-3-meth­oxy­phenyl­methyl)-5,5-di­methyl­cyclo­hex-2-enone

**DOI:** 10.1107/S2056989018006941

**Published:** 2018-05-15

**Authors:** Agnese Stikute, Karina Skestere, Inese Mierina, Anatoly Mishnev, Mara Jure

**Affiliations:** a Institute of Technology of Organic Chemistry, Faculty of Materials Science and Applied Chemistry, Riga Technical University, P. Valdena Str. 3/7, Riga, LV-1048, Latvia; b Latvian Institute of Organic Synthesis, Aizkraukles Str. 21, Riga, LV-1006, Latvia

**Keywords:** crystal structure, aryl­methyl dimedone, aryl­methyl 1,3-cyclo­hexa­nedione, aryl­methyl 3-hy­droxy­cyclo­hex-2-enone

## Abstract

In the title compound, C_16_H_20_O_4_, a new starting compound for the synthesis of various heterocycles, the partially saturated six-membered ring adopts a sofa conformation. An intra­molecular O—H⋯O hydrogen bond is observed in the guaiacol residue. In the crystal, mol­ecules are assembled into a sheet structure parallel to the *ab* plane *via* O—H⋯O hydrogen bonds. The hydrogen-bond pattern is described by an *R*_4_^4^(28) graph-set motif. The sheets are further linked by C—H⋯O hydrogen bonds into a three-dimensional network.

## Abstract 2

In the title dimedone derivative, the 4-hy­droxy-3-meth­oxy­benzyl substituent adopts a sofa conformation. In the crystal, mol­ecules are assembled into a sheet structure parallel to the *ab* plane *via* O—H⋯O hydrogen bonds.

## Chemical context   

Cyclic 2-aryl­methyl-1,3-diketones attract inter­est as valuable inter­mediates for organic chemistry. A few of the latest examples of these cyclo­hexa­nedione derivatives have been used as starting compounds for the synthesis of various heterocycles [*e.g*. tetra­hydro­benzo­furan­ones (Yoshida *et al.*, 2010 ▸) or tetra­hydro-1*H*-xanthen-1-ones (Sudheendran *et al.*, 2012 ▸)], as well as carbocycles, *e.g.* analogues of Wieland–Miesher and Hajos–Parrish ketones (Xu *et al.*, 2013 ▸).
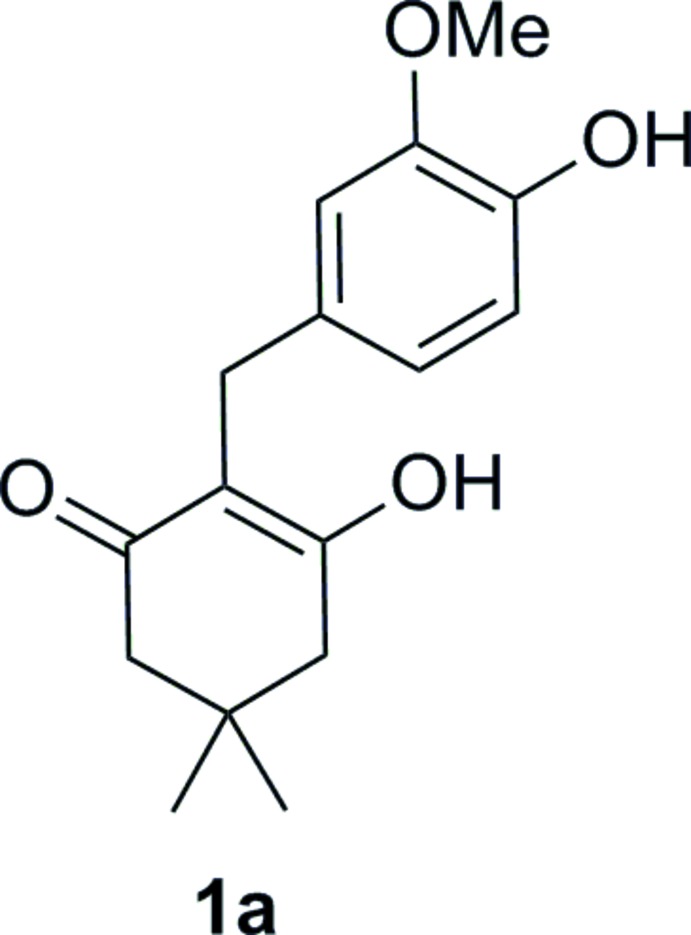


## Structural commentary   

**Figure 1:**
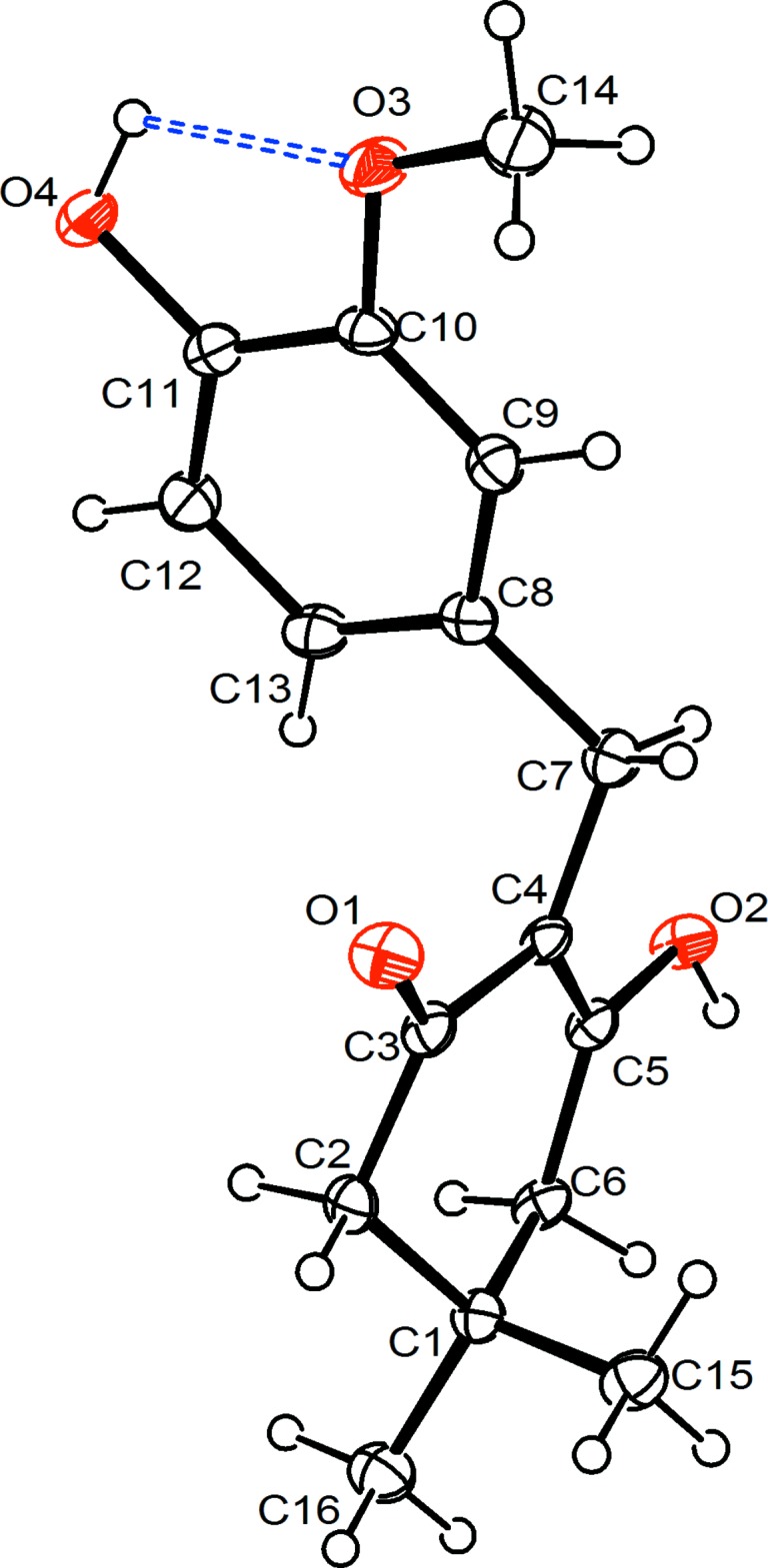
The mol­ecular structure of the title compound, with the atom-numbering scheme and 50% probability displacement ellipsoids. The intra­molecular hydrogen bond is shown as a double-dashed line.

Fig. 1 ▸ shows the mol­ecular structure of the title compound, which exhibits an intra­molecular O—H⋯O hydrogen bond (Table 1 ▸). In crystalline state, the mol­ecules assume the enol tautomeric form, **1a**. In the dimedone fragment, the bond distances reflect the effect of conjugation in the flat fragment O1=C3—C4=C5—O2. The double bonds, O1=C3 and C4=C5, are elongated [1.246 (2) and 1.357 (3) Å, respectively], while the single bond C3—C4 is shortened [1.447 (3) Å] as compared with standard double and single bonds (Allen *et al.*, 1987 ▸). The general shape of the mol­ecule is characterized by the torsion angles C3—C4—C7—C8 = −62.8 (2)° and C4—C7—C8—C9 = 152.2 (2)°, thus exhibiting an extended conformation. The partially saturated C1–C6 ring adopts a sofa conformation. The distance of atom C1 from the mean plane formed by atoms C2–C6 is 0.612 (3) Å. The dihedral angle between the mean plane of the C1–C6 ring and the C8–C13 benzene ring is 75.69 (6)°.

## Supra­molecular features   

**Figure 2:**
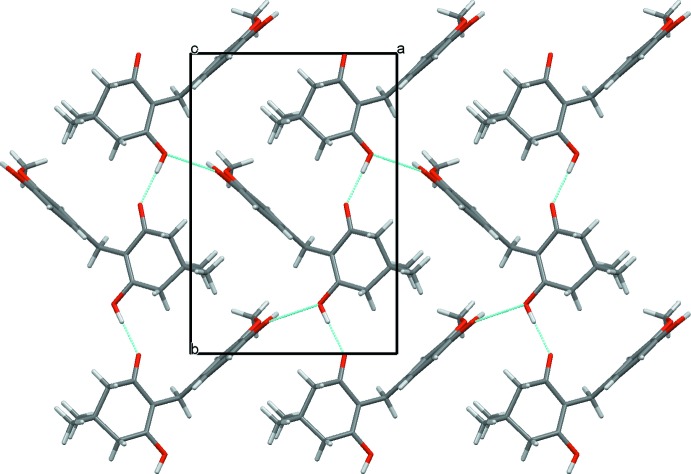
A packing diagram of the title compound, viewed along the *c* axis. O—H⋯O hydrogen bonds are shown as dashed lines. For clarity weak C—H⋯O bonds are not depicted.

In the crystal, the mol­ecules are assembled into a sheet structure parallel to the *ab* plane *via* O—H⋯O hydrogen bonds (Table 1 ▸). The hydrogen-bonding pattern in the sheet is described by an 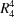
(28) graph-set motif (Fig. 2 ▸). Furthermore, weak C—H⋯O hydrogen bonds join the sheets into a three-dimensional network (Table 1 ▸).

## Database survey   

A search of the Cambridge Structural Database (Version 5.39, last update February 2018; Groom *et al.*, 2016 ▸) gave 76 structures of 3-hy­droxy-5,5-di­methyl­cyclo­hex-2-enone derivatives. The closest structures are 2-(naphthalen-1-ylmeth­yl)- and 2-(3-chloro­phen­yl)methyl-substituted dimedones (NIHTEE and NIHTII, respectively; Ramachary & Kishor, 2007 ▸).

## Antiradical activity against free radicals   

Compound **1** demonstrates notable anti­radical activity against free radicals. Free radical tests were realized according to the procedures described previously (Mierina *et al.*, 2017 ▸). 1,1-Diphenyl-2-picrylhydrazyl test: inhibition, when molar ratio of the compound and free radical is 1:1, was 93.3±2.5%; IC_50_ was 23.0±0.6 µM (starting concentration of free radical was 100 µM). Galvinoxyl test: inhibition was 82.3±1.0% and IC_50_ – 20.3±2.0 µM.

## Synthesis and crystallization   

**Figure 3:**
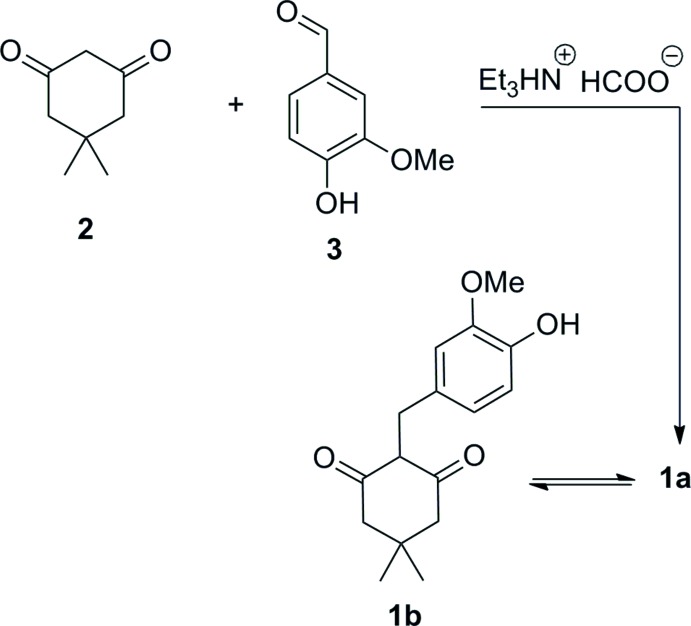
Reaction scheme for the title compound (**1a**) and its tautomer (**1 b**).

3-Hy­droxy-2-(4-hy­droxy-3-meth­oxy­phenyl­meth­yl)-5,5-di­methyl­cyclo­hex-2-enone (**1a**) was synthesized according to the reaction scheme in Fig. 3 ▸. Formic acid (3.6 ml) was added to a solution of dimedone **2** (500 mg, 3.6 mmol) and vanillin **3** (543 mg, 3.6 mmol) in tri­ethyl­amine (5.5 ml) while cooling in an ice-bath. The reaction mixture was then heated at 413 K for 5 h, followed by cooling to room temperature, pouring into ice (700–800 ml) and filtering the formed solid. The solid material was purified by crystallization from chloro­form leading to the target compound **1a** (615 mg, 62%) with m.p. 466–468 K. Single crystals were obtained from a methanol solution. IR (KBr) ν, cm^−1^: 3470, 2935, 2645, 1580, 1515, 1375, 1250, 1230, 1200, 1040.

The enol form, **1a**, was observed exclusively in a DMSO solution. ^1^H NMR for compound **1a** (300 MHz, DMSO-*d_6_*) δ, ppm: 10.71–10.08 (1H, *brs*, OH), 8.68–8.37 (1H, *brs*, OH), 6.68 (1H, *s*, H^Ar^), 6.59 (1H, *d*, *J* = 7.7 Hz, H^Ar^), 6.50 (1H, *d*, *J* = 7.7 Hz, H^Ar^), 3.68 (3H, *s*, OMe), 3.41 (2H, *brs*, CH_2_Ar, overlapping with H_2_O signal), 2.34–2.13 (4H, *brs*, 2CH_2_), 0.98 (6H, *s*, 2Me). ^13^C NMR for compound **1a** (75 MHz, DMSO-*d_6_*) δ, ppm: 147.1, 144.1, 132.7, 120.2, 115.0, 113.3, 112.5, 55.5, 31.7, 28.0, 26.5. Mixture of keto–enol tautomers (**1a** and **1b**) was observed in a chloro­form solution. The ratio of enol **1a** and ketone **1b** was 1.35:1 (at room temperature). ^1^H NMR for compound **1a** (300 MHz, CDCl_3_) δ, ppm: 6.84–6.63 (3H, *m*, H^Ar^), (2H, *brs*, 2OH), 3.82 (3H, *s*, OMe), 3.61 (2H, *s*, CH_2_Ar), 2.33–2.29 (4H, *brs*, 2CH_2_), 1.07 (6H, *s*, 2Me). ^1^H NMR for compound **1b** (300 MHz, CDCl_3_) δ, ppm: 6.84–6.63 (3H, *m*, H^Ar^), 5.62–5.68 (1H, *brs*, OH), 3.86 (3H, *s*, OMe), 3.56 (1H, *t*, *J* = 5.4 Hz, CHCH_2_), 3.11 (2H, *d*, *J* = 5.4 Hz, CHCH_2_), 2.65 (2H, *d*, *J* = 13.4 Hz, H^a^ from CH_2_), 2.44 (2H, *d*, *J* = 13.4 Hz, H^b^ from CH_2_), 1.16 (3H, *s*, Me), 0.82 (3H, s, Me).

## Refinement   

Crystal data, data collection and structure refinement details are summarized in Table 2 ▸. Hydrogen atoms bonded to O atoms were refined freely. Other H atoms were included in the refinement at geometrically calculated positions with C—H = 0.93–0.97 Å and treated as riding with *U*_iso_(H) = 1.2*U*_eq_(C) or 1.5*U*_eq_(C-meth­yl).

## Supplementary Material

Crystal structure: contains datablock(s) I. DOI: 10.1107/S2056989018006941/is5495sup1.cif

Structure factors: contains datablock(s) I. DOI: 10.1107/S2056989018006941/is5495Isup2.hkl

Supporting information file. DOI: 10.1107/S2056989018006941/is5495Isup3.cml

CCDC reference: 1841730

Additional supporting information:  crystallographic information; 3D view; checkCIF report

## Figures and Tables

**Table 1:** Hydrogen-bond geometry (Å, °)

*D*—H⋯*A*	*D*—H	H⋯*A*	*D*⋯*A*	*D*—H⋯*A*
C2—H2*A*⋯O4^i^	0.97	2.49	3.417 (3)	161
C14—H14*C*⋯O1^ii^	0.96	2.49	3.247 (3)	136
O2—H2⋯O1^iii^	0.88 (3)	1.74 (3)	2.586 (2)	161 (3)
O4—H4⋯O3	0.94 (4)	2.10 (4)	2.638 (2)	115 (3)
O4—H4⋯O2^iv^	0.94 (4)	2.11 (4)	2.919 (2)	142 (3)

Symmetry codes: (i) 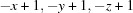; (ii) 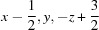; (iii) 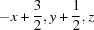; (iv) 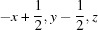.

**Table 2:** Experimental details

Crystal data	
Chemical formula	C_16_H_20_O_4_
*M*_r_	276.32
Crystal system, space group	Orthorhombic, *P* *b* *c* *a*
Temperature (K)	190
*a*, *b*, *c* (Å)	9.3504 (3), 13.6265 (4), 22.8790 (9)
*V* (Å^3^)	2915.09 (17)
*Z*	8
Radiation type	Mo *K*α
μ (mm^−1^)	0.09
Crystal size (mm)	0.32 × 0.17 × 0.12

Data collection	
Diffractometer	Bruker KappaCCD
No. of measured, independent and observed [*I* > 2σ(*I*)] reflections	6082, 3295, 2149
*R*_int_	0.057
(sin θ/λ)_max_ (Å^−1^)	0.649

Refinement	
*R*[*F* ^2^ > 2σ(*F* ^2^)], *wR*(*F* ^2^), *S*	0.059, 0.131, 1.04
No. of reflections	3295
No. of parameters	192
H-atom treatment	H atoms treated by a mixture of independent and constrained refinement
Δρ_max_, Δρ_min_ (e Å^−3^)	0.21, −0.19

Computer programs: *COLLECT* (Bruker, 2001 ▸), *SCALEPACK* (Otwinowski & Minor, 1997 ▸), *DENZO* (Otwinowski & Minor, 1997 ▸), *SIR2004* (Burla *et al.*, 2005 ▸), *SHELXL2017* (Sheldrick, 2015 ▸), *ORTEP-3 for Windows* (Farrugia, 2012 ▸) and *publCIF* (Westrip, 2010 ▸).

## supplementary crystallographic information

### Crystal data

**Table:**

C_16_H_20_O_4_	*D*_x_ = 1.259 Mg m^−^^3^
*M**_r_* = 276.32	Mo *K*α radiation, λ = 0.71073 Å
Orthorhombic, *P**b**c**a*	Cell parameters from 32401 reflections
*a* = 9.3504 (3) Å	θ = 1.0–27.5°
*b* = 13.6265 (4) Å	µ = 0.09 mm^−^^1^
*c* = 22.8790 (9) Å	*T* = 190 K
*V* = 2915.09 (17) Å^3^	Block, colourless
*Z* = 8	0.32 × 0.17 × 0.12 mm
*F*(000) = 1184	

### Data collection

**Table:**

Bruker KappaCCD diffractometer	*R*_int_ = 0.057
CCD scans	θ_max_ = 27.5°, θ_min_ = 2.8°
6082 measured reflections	*h* = −12→12
3295 independent reflections	*k* = −17→17
2149 reflections with *I* > 2σ(*I*)	*l* = −29→29

### Refinement

**Table:**

Refinement on *F*^2^	0 restraints
Least-squares matrix: full	Hydrogen site location: mixed
*R*[*F*^2^ > 2σ(*F*^2^)] = 0.059	H atoms treated by a mixture of independent and constrained refinement
*wR*(*F*^2^) = 0.131	*w* = 1/[σ^2^(*F*_o_^2^) + (0.0489*P*)^2^ + 1.0327*P*] where *P* = (*F*_o_^2^ + 2*F*_c_^2^)/3
*S* = 1.04	(Δ/σ)_max_ = 0.006
3295 reflections	Δρ_max_ = 0.21 e Å^−^^3^
192 parameters	Δρ_min_ = −0.19 e Å^−^^3^

### Special details

**Table:**

Geometry. All esds (except the esd in the dihedral angle between two l.s. planes) are estimated using the full covariance matrix. The cell esds are taken into account individually in the estimation of esds in distances, angles and torsion angles; correlations between esds in cell parameters are only used when they are defined by crystal symmetry. An approximate (isotropic) treatment of cell esds is used for estimating esds involving l.s. planes.

### Fractional atomic coordinates and isotropic or equivalent isotropic displacement parameters (Å^2^)

**Table:**

	*x*	*y*	*z*	*U*_iso_*/*U*_eq_	
O1	0.75371 (16)	0.50507 (9)	0.63597 (7)	0.0331 (4)	
O2	0.62541 (15)	0.83466 (10)	0.63025 (7)	0.0287 (4)	
O3	0.16966 (16)	0.39470 (10)	0.68561 (7)	0.0367 (4)	
O4	0.13650 (15)	0.39579 (10)	0.57111 (7)	0.0319 (4)	
C1	0.9683 (2)	0.71070 (12)	0.58699 (9)	0.0224 (4)	
C2	0.8992 (2)	0.60988 (13)	0.57748 (9)	0.0256 (5)	
H2A	0.864618	0.605845	0.537577	0.031*	
H2B	0.971321	0.559458	0.582589	0.031*	
C3	0.7777 (2)	0.58996 (13)	0.61829 (9)	0.0230 (5)	
C4	0.6838 (2)	0.66925 (13)	0.63552 (9)	0.0213 (4)	
C5	0.7173 (2)	0.76168 (13)	0.61827 (9)	0.0216 (4)	
C6	0.8495 (2)	0.78837 (13)	0.58489 (9)	0.0243 (5)	
H6A	0.886941	0.849470	0.600375	0.029*	
H6B	0.823687	0.799694	0.544389	0.029*	
C7	0.5558 (2)	0.64569 (13)	0.67303 (10)	0.0277 (5)	
H7A	0.508367	0.706600	0.683286	0.033*	
H7B	0.589063	0.615710	0.709003	0.033*	
C8	0.4470 (2)	0.57763 (13)	0.64472 (9)	0.0232 (5)	
C9	0.3616 (2)	0.51732 (13)	0.68030 (9)	0.0258 (5)	
H9	0.374309	0.517992	0.720617	0.031*	
C10	0.2589 (2)	0.45705 (13)	0.65589 (9)	0.0252 (5)	
C11	0.2391 (2)	0.45584 (13)	0.59560 (9)	0.0244 (5)	
C12	0.3230 (2)	0.51344 (15)	0.56037 (10)	0.0300 (5)	
H12	0.310764	0.512153	0.520037	0.036*	
C13	0.4265 (2)	0.57391 (14)	0.58529 (10)	0.0287 (5)	
H13	0.483160	0.612663	0.561127	0.034*	
C14	0.1966 (3)	0.37943 (17)	0.74571 (11)	0.0469 (7)	
H14A	0.293311	0.357396	0.750859	0.070*	
H14B	0.131964	0.330679	0.760536	0.070*	
H14C	0.182851	0.439830	0.766563	0.070*	
C15	1.0448 (2)	0.71292 (14)	0.64588 (10)	0.0317 (5)	
H15A	0.977325	0.700158	0.676564	0.048*	
H15B	1.087200	0.776352	0.651680	0.048*	
H15C	1.118201	0.663631	0.646450	0.048*	
C16	1.0760 (2)	0.73067 (15)	0.53816 (10)	0.0345 (5)	
H16A	1.115861	0.795077	0.543051	0.052*	
H16B	1.028672	0.726735	0.500998	0.052*	
H16C	1.151155	0.682745	0.539763	0.052*	
H2	0.664 (3)	0.892 (2)	0.6238 (13)	0.079 (10)*	
H4	0.085 (4)	0.368 (3)	0.6024 (16)	0.114 (14)*	

### Atomic displacement parameters (Å^2^)

**Table:**

	*U*^11^	*U*^22^	*U*^33^	*U*^12^	*U*^13^	*U*^23^
O1	0.0372 (8)	0.0110 (6)	0.0510 (10)	−0.0009 (6)	0.0078 (8)	0.0033 (6)
O2	0.0240 (8)	0.0128 (7)	0.0493 (10)	0.0022 (6)	−0.0014 (7)	0.0001 (6)
O3	0.0393 (9)	0.0406 (9)	0.0304 (9)	−0.0196 (7)	0.0011 (7)	0.0042 (7)
O4	0.0283 (8)	0.0362 (8)	0.0312 (9)	−0.0096 (7)	−0.0035 (7)	−0.0033 (7)
C1	0.0241 (10)	0.0168 (9)	0.0262 (11)	−0.0004 (8)	0.0006 (9)	0.0004 (8)
C2	0.0287 (11)	0.0172 (9)	0.0308 (12)	0.0006 (8)	0.0033 (9)	−0.0035 (8)
C3	0.0253 (11)	0.0149 (9)	0.0288 (12)	−0.0019 (8)	−0.0012 (9)	−0.0016 (8)
C4	0.0199 (10)	0.0145 (9)	0.0297 (12)	−0.0023 (8)	−0.0019 (9)	−0.0035 (8)
C5	0.0207 (11)	0.0163 (9)	0.0278 (12)	0.0010 (8)	−0.0047 (8)	−0.0014 (8)
C6	0.0241 (10)	0.0151 (9)	0.0336 (12)	−0.0028 (8)	−0.0038 (9)	0.0029 (8)
C7	0.0306 (12)	0.0160 (9)	0.0366 (14)	−0.0026 (9)	0.0042 (10)	−0.0034 (8)
C8	0.0222 (10)	0.0164 (9)	0.0310 (12)	0.0031 (8)	0.0031 (9)	0.0003 (8)
C9	0.0283 (11)	0.0233 (10)	0.0260 (12)	0.0005 (9)	0.0014 (9)	0.0000 (8)
C10	0.0236 (10)	0.0198 (9)	0.0322 (12)	−0.0023 (9)	0.0060 (9)	0.0024 (8)
C11	0.0207 (10)	0.0197 (9)	0.0328 (12)	0.0011 (8)	−0.0013 (9)	−0.0003 (9)
C12	0.0330 (12)	0.0300 (11)	0.0271 (13)	−0.0028 (10)	−0.0005 (10)	0.0037 (9)
C13	0.0279 (11)	0.0222 (10)	0.0359 (13)	−0.0035 (9)	0.0038 (10)	0.0061 (9)
C14	0.0604 (17)	0.0503 (14)	0.0300 (14)	−0.0262 (13)	0.0077 (12)	0.0032 (11)
C15	0.0263 (11)	0.0273 (11)	0.0414 (14)	0.0024 (9)	−0.0045 (10)	−0.0003 (10)
C16	0.0333 (12)	0.0270 (11)	0.0432 (15)	−0.0024 (10)	0.0077 (10)	0.0000 (10)

### Geometric parameters (Å, º)

**Table:**

O1—C3	1.246 (2)	C7—H7A	0.9700
O2—C5	1.342 (2)	C7—H7B	0.9700
O2—H2	0.88 (3)	C8—C13	1.374 (3)
O3—C10	1.371 (2)	C8—C9	1.406 (3)
O3—C14	1.413 (3)	C9—C10	1.381 (3)
O4—C11	1.380 (2)	C9—H9	0.9300
O4—H4	0.94 (4)	C10—C11	1.392 (3)
C1—C15	1.526 (3)	C11—C12	1.371 (3)
C1—C16	1.529 (3)	C12—C13	1.394 (3)
C1—C2	1.534 (3)	C12—H12	0.9300
C1—C6	1.534 (3)	C13—H13	0.9300
C2—C3	1.495 (3)	C14—H14A	0.9600
C2—H2A	0.9700	C14—H14B	0.9600
C2—H2B	0.9700	C14—H14C	0.9600
C3—C4	1.447 (3)	C15—H15A	0.9600
C4—C5	1.357 (3)	C15—H15B	0.9600
C4—C7	1.507 (3)	C15—H15C	0.9600
C5—C6	1.498 (3)	C16—H16A	0.9600
C6—H6A	0.9700	C16—H16B	0.9600
C6—H6B	0.9700	C16—H16C	0.9600
C7—C8	1.521 (3)		
			
C5—O2—H2	111 (2)	C13—C8—C9	118.18 (18)
C10—O3—C14	117.73 (17)	C13—C8—C7	122.47 (18)
C11—O4—H4	107 (2)	C9—C8—C7	119.33 (19)
C15—C1—C16	109.41 (17)	C10—C9—C8	120.5 (2)
C15—C1—C2	109.91 (16)	C10—C9—H9	119.7
C16—C1—C2	109.48 (16)	C8—C9—H9	119.7
C15—C1—C6	110.69 (16)	O3—C10—C9	126.20 (19)
C16—C1—C6	109.34 (16)	O3—C10—C11	113.78 (17)
C2—C1—C6	107.99 (16)	C9—C10—C11	120.02 (18)
C3—C2—C1	113.20 (15)	C12—C11—O4	119.9 (2)
C3—C2—H2A	108.9	C12—C11—C10	119.98 (19)
C1—C2—H2A	108.9	O4—C11—C10	120.14 (18)
C3—C2—H2B	108.9	C11—C12—C13	119.7 (2)
C1—C2—H2B	108.9	C11—C12—H12	120.2
H2A—C2—H2B	107.8	C13—C12—H12	120.2
O1—C3—C4	119.70 (18)	C8—C13—C12	121.57 (19)
O1—C3—C2	120.54 (17)	C8—C13—H13	119.2
C4—C3—C2	119.71 (16)	C12—C13—H13	119.2
C5—C4—C3	118.27 (18)	O3—C14—H14A	109.5
C5—C4—C7	123.16 (17)	O3—C14—H14B	109.5
C3—C4—C7	118.55 (16)	H14A—C14—H14B	109.5
O2—C5—C4	118.71 (17)	O3—C14—H14C	109.5
O2—C5—C6	116.90 (15)	H14A—C14—H14C	109.5
C4—C5—C6	124.37 (17)	H14B—C14—H14C	109.5
C5—C6—C1	114.44 (15)	C1—C15—H15A	109.5
C5—C6—H6A	108.7	C1—C15—H15B	109.5
C1—C6—H6A	108.7	H15A—C15—H15B	109.5
C5—C6—H6B	108.7	C1—C15—H15C	109.5
C1—C6—H6B	108.7	H15A—C15—H15C	109.5
H6A—C6—H6B	107.6	H15B—C15—H15C	109.5
C4—C7—C8	114.73 (17)	C1—C16—H16A	109.5
C4—C7—H7A	108.6	C1—C16—H16B	109.5
C8—C7—H7A	108.6	H16A—C16—H16B	109.5
C4—C7—H7B	108.6	C1—C16—H16C	109.5
C8—C7—H7B	108.6	H16A—C16—H16C	109.5
H7A—C7—H7B	107.6	H16B—C16—H16C	109.5
			
C15—C1—C2—C3	67.9 (2)	C3—C4—C7—C8	−62.8 (2)
C16—C1—C2—C3	−171.93 (17)	C4—C7—C8—C13	−29.1 (3)
C6—C1—C2—C3	−53.0 (2)	C4—C7—C8—C9	152.22 (17)
C1—C2—C3—O1	−146.67 (19)	C13—C8—C9—C10	−0.8 (3)
C1—C2—C3—C4	35.9 (3)	C7—C8—C9—C10	177.99 (17)
O1—C3—C4—C5	176.34 (19)	C14—O3—C10—C9	−9.4 (3)
C2—C3—C4—C5	−6.2 (3)	C14—O3—C10—C11	170.34 (19)
O1—C3—C4—C7	−2.0 (3)	C8—C9—C10—O3	179.63 (18)
C2—C3—C4—C7	175.47 (18)	C8—C9—C10—C11	−0.1 (3)
C3—C4—C5—O2	175.08 (17)	O3—C10—C11—C12	−178.86 (17)
C7—C4—C5—O2	−6.6 (3)	C9—C10—C11—C12	0.9 (3)
C3—C4—C5—C6	−3.2 (3)	O3—C10—C11—O4	0.3 (3)
C7—C4—C5—C6	175.11 (19)	C9—C10—C11—O4	−179.93 (16)
O2—C5—C6—C1	163.97 (17)	O4—C11—C12—C13	−179.95 (17)
C4—C5—C6—C1	−17.7 (3)	C10—C11—C12—C13	−0.8 (3)
C15—C1—C6—C5	−76.1 (2)	C9—C8—C13—C12	0.9 (3)
C16—C1—C6—C5	163.26 (17)	C7—C8—C13—C12	−177.81 (18)
C2—C1—C6—C5	44.2 (2)	C11—C12—C13—C8	−0.1 (3)
C5—C4—C7—C8	118.9 (2)		

### Hydrogen-bond geometry (Å, º)

**Table:**

*D*—H···*A*	*D*—H	H···*A*	*D*···*A*	*D*—H···*A*
C2—H2*A*···O4^i^	0.97	2.49	3.417 (3)	161
C14—H14*C*···O1^ii^	0.96	2.49	3.247 (3)	136
O2—H2···O1^iii^	0.88 (3)	1.74 (3)	2.586 (2)	161 (3)
O4—H4···O3	0.94 (4)	2.10 (4)	2.638 (2)	115 (3)
O4—H4···O2^iv^	0.94 (4)	2.11 (4)	2.919 (2)	142 (3)

Symmetry codes: (i) −*x*+1, −*y*+1, −*z*+1; (ii) *x*−1/2, *y*, −*z*+3/2; (iii) −*x*+3/2, *y*+1/2, *z*; (iv) −*x*+1/2, *y*−1/2, *z*.
